# Translation, cross-cultural adaptation and validation of the Work Role Functioning Questionnaire 2.0 into Brazilian Portuguese in a general population

**DOI:** 10.1186/s12955-022-01924-y

**Published:** 2022-02-03

**Authors:** Josane Soares Pinto Melo, Almir Vieira Dibai-Filho, Adriana Sousa Rêgo, Daniel Santos Rocha, Isabel Aparecida Porcatti de Walsh, Rudys Rodolfo de Jesus Tavarez, Maria Claudia Gonçalves, Karen Larissa Brito Damasceno, Cid André Fidelis de Paula Gomes, Daniela Bassi-Dibai

**Affiliations:** 1 Postgraduate Program in Programs Management and Health Services, Universidade Ceuma, Rua Josué Montello, 1, Jardim Renascença, São Luís, MA 65075-120 Brazil; 2 Postgraduate Program in Physical Education, Universidade Federal do Maranhão, São Luís, MA Brazil; 3 Department of Physical Therapy, Universidade Ceuma, São Luís, MA Brazil; 4 Postgraduate Program in Physical Therapy, Universidade Federal do Triângulo Mineiro, Uberaba, MG Brazil; 5 Postgraduate Program in Dentistry, Universidade Ceuma, São Luís, MA Brazil; 6 Postgraduate Program in Environment, Universidade Ceuma, São Luís, MA Brazil; 7 Postgraduate Program in Rehabilitation Sciences, Universidade Nove de Julho, São Paulo, SP Brazil

**Keywords:** Work, Role functioning, Questionnaire, Occupational health, Cross-cultural, Validation studies

## Abstract

**Background:**

The Work Role Functioning Questionnaire 2.0 (WRFQ 2.0) is an instrument that measures the difficulties perceived by workers in meeting work demands, given their physical or emotional health, but it has not yet been adapted for Brazil. Thus, this study aimed to translate, cross-culturally adapt and assess the psychometric properties of the WRFQ 2.0 into Brazilian Portuguese.

**Methods:**

This is an observational study. Initially, translation and cross-cultural adaptation into Brazilian Portuguese was carried out. After that, this version was submitted to an evaluation of its internal structure. The internal consistency and test–retest reliability were assessed. To determine the construct validity, Spearman's correlation coefficient (rho) was used to determine the magnitude of correlation between the WRFQ 2.0 and the Work Ability Index (WAI), Numerical Pain Rating Scale (NPRS) and Self -Estimated Functional Inability because of Pain (SEFIP-work).

**Results:**

The internal structure with five domains and 27 items presented adequate fit indices for the Brazilian version of the WRFQ 2.0. Adequate correlations of the five domains of the WRFQ 2.0 with the NPRS, WAI and SEFIP-work were found (rho ranged between 0.145 and 0.338). The test–retest reliability of the WRFQ 2.0 ranged from substantial to excellent (intraclass correlation coefficient ≥ 0.785) and internal consistency was adequate (Cronbach's alpha ≥ 0.852).

**Conclusion:**

The Brazilian Portuguese version of the WRFQ 2.0 presents valid internal structure with five domains and 27 items, adequate construct based on correlations with other instruments, and acceptable test–retest reliability and internal consistency.

**Supplementary Information:**

The online version contains supplementary material available at 10.1186/s12955-022-01924-y.

## Background

Occupational diseases are caused by a set of interrelated factors, such as demand for excessive force, forced posture, repetition of the same movement for long periods and mechanical compression. In addition to these factors, aspects related to the organization of work can also compromise the health of workers; for example, long hours, absence of periodic and spontaneous breaks, high productivity requirement, intense work pace, stressful environment, high demand for attention to avoid errors and submission to permanent monitoring [1].

In this context, assessment instruments based on self-reporting have been commonly used for occupational health assessment. However, most of these instruments are specific to assess work-related disability with a focus on musculoskeletal dysfunction and pain or postural and biomechanical aspects [2, 3].

The Work Role Functioning Questionnaire (WRFQ) was developed in English in 2002, and has been translated into more than ten languages to measure the impact of chronic diseases on the performance of daily work activities [4, 5]. It is widely used because it was developed to represent a wide variety of work demands, as well as health problems. The cross-culturally adapted versions have adequate measurement properties, including the Brazilian Portuguese version [6].

During the translation and adaptation of the WRFQ into the Dutch language, the need for adjustments to the instrument was identified. Based on this scientific initiative, it was possible to formulate new items to better reflect the changes in work in recent decades. A new version of the WRFQ, called WRFQ 2.0, was then created, with 27 items (inclusion of five new items) organized into five domains: (1) work scheduling demands, (2) output demands, (3) physical demands, (4) mental and social demands and, (5) flexible demands [5].

The purpose of the WRFQ 2.0 is to measure the difficulties perceived by workers in meeting work demands, given their physical or emotional health. This instrument is available in the Dutch [5], Norwegian, Danish [7] and Persian languages [8]. In the Dutch version [5], the authors reported adequate internal structure with 4 domains, in addition to adequate construct (moderate correlations with Endicott Work Productivity Scale) and acceptable reliability and internal consistency (intraclass correlation coefficient [ICC] ranging from 0.29 to 0.82 and Cronbach's alphas ≥ 0.91, respectively). Subsequently, another study identified the internal structure with 5 domains that was also adequate [9].

The Norwegian and Danish versions [7] showed incomplete cross-cultural adaptation due to the absence of important psychometric properties, only internal consistency was assessed (Cronbach's alpha ≥ 0.79). In the Persian version [8], adequate internal structure with 4 domains was observed, in addition to acceptable reliability and internal consistency (ICC ranging from 0.87 to 0.96 and Cronbach's alpha ≥ 0.87, respectively). The Persian version did not assess the construct through correlation with instruments already validated for the country.

To date, the WRFQ 2.0 is not scientifically supported for use in the Brazilian population. In this context and considering the evaluative importance of this instrument, the aim of this study was to translate, cross-culturally adapt and assess the psychometric properties of the WRFQ 2.0 into Brazilian Portuguese.

## Methods

### Study design and setting

This was an observational study to assess the psychometric properties of the WRFQ 2.0, performed according to the Guidelines for the Process of Cross-cultural Adaptation of Self-Report Measures [10] and the Consensus-based Standards for the Selection of Health Measurement Instruments (COSMIN) [11]. The authorization for cross-cultural adaptation into Brazilian Portuguese of the WRFQ 2.0 was granted via email by the main author of the original study (Dr. Femke I. Abma).

The present study was carried out in two phases: 1) translation and adaptation of the WRFQ 2.0 with analysis of the pre-final version into Brazilian Portuguese, and 2) validation and reliability of the final version of the WRFQ 2.0 cross-culturally adapted into Brazilian Portuguese.

This study was carried out by face-to-face collection in health units in São Luís (Maranhão, northeastern Brazil), as well as through the online platform Google Forms (Mountain View, CA, USA). The study procedures were approved by the research ethics committee of the Universidade Ceuma (opinion number 3,779,579). Recruitment of the volunteers took place through verbal contact, posters and social media. All volunteers included in the study validated their participation by signing or electronically consenting on the free and informed consent form.

### Translation and cross-cultural adaptation

The WRFQ 2.0 translation and cross-cultural adaptation process into Brazilian Portuguese followed the criteria of Beaton et al. [10] and was carried out in five stages, described below:

1. Translation: two independent translators (a physiotherapist with 11 years of experience in the field and an English teacher with experience in translations for 22 years without technical knowledge of subjects in the health area), both with Brazilian Portuguese as their mother tongue and fluency in English, translated the original version of the WRFQ 2.0 into Brazilian Portuguese.
2. Synthesis of translations: after discussions and revisions, the two translators, under observation by one of the researchers, synthesized the two versions of the questionnaire translated independently and produced a single version of the WRFQ 2.0 in a consensual manner.
3. Back-translation: two independent translators (without technical knowledge of subjects in the health area), both with English as their mother tongue, fluent in Portuguese and residing in Brazil, translated the Brazilian Portuguese version of the WRFQ 2.0 back to English, without any prior knowledge of the original version of the questionnaire.
4. Analysis by a committee of experts: five specialists from the occupational health field, together with the four translators involved in the project, reviewed all translated and back-translated versions to correct possible discrepancies; achieving a pre-final version of the WRFQ 2.0 in an agreed manner between all committee members.
5. Pre-final version test: the pre-final version of the WRFQ 2.0 was applied to 30 workers with Brazilian Portuguese as their mother tongue, able to read and complete the questionnaire and, upon completion, establish their understanding of the pre-final version of the WRFQ 2.0 by checking a checkbox containing the answers “yes” and “no” for each item of the questionnaire. A level of understanding higher than 80% of the sample was established as an acceptability criterion for the pre-final version [12].

### Study sample

To calculate the sample size, the COSMIN recommendation of multiplying the number of items in the questionnaire by 7 to perform the factor analysis was used [11]. Therefore, as the WRFQ 2.0 has 27 items, the minimum sample size was 189 participants.

Active workers with at least six months in the same job included in the study [13] were aged 18 years or older and able to read and write in Brazilian Portuguese. The participants excluded from the study were workers who reported some diagnosis of cognitive and/or psychiatric illness that impeded proper understanding of the research instruments, as well as workers who were away from the work environment.

To perform the test of the pre-final version of the WRFQ 2.0, the sample consisted of 30 participants [10]. Participants answered the WRFQ 2.0 and indicated whether or not they understood each item in the questionnaire. For the reliability analyses, the WRFQ 2.0 was applied to a sub-sample containing 35 participants at two different times, with an interval of seven days between assessments. In order to verify the psychometric properties of the instrument, the final cross-culturally adapted version of the WRFQ 2.0 was then applied to 197 workers.

### Work Role Functioning Questionnaire 2.0 (WRFQ 2.0)

The WRFQ 2.0 is an instrument that measures the difficulties perceived by workers in meeting work demands, given their physical or emotional health. It was originally developed by Abma et al. [5] and consists of 27 items divided into five domains: work scheduling demands (items 1–4), output demands (items 5–10), physical demands (items 11–15), mental and social demands (items 16–22), and flexible demands (items 23–27). For each item, it is possible to mark “all of the time” (score 0) to “none of the time” (score 4). The total score is transformed into a percentage (0–100%), and the lower the score, the greater the difficulties/poor work functioning.

### Other assessments

In addition to collecting information related to the participants' personal and occupational characteristics, we used three tools with suitable measurement properties for the Brazilian population for construct validity.

We used the Work Ability Index (WAI), instrument with suitable measurement properties for Brazilian Portuguese [14]. This instrument is composed of several questions, considering illnesses, physical and mental demands of work, forming seven domains, namely: (1) current work ability compared to the best in life (score from 0 to 10), (2) ability to work in relation to the demands of the job (score from 2 to 10), (3) number of current diseases diagnosed by a physician (score from 1 to 7), (4) estimated loss to work due to illness (score 1–6), (5) absenteeism due to illnesses in the last year (score from 1 to 5), (6) proper prognosis of work ability in two years (score from 1 to 7), and (7) mental resources (score from 1 to 4).

The Numerical Pain Rating Scale (NPRS) was used to measure pain intensity, in which 11 points (from 0 to 10) are possible, with 0 indicating “no pain” and 10 representing “the most unbearable pain”. The individual also identifies the maximum pain intensity at the time. This instrument presents suitable measurement properties for Portuguese [15].

The Self-Estimated Functional Inability because of Pain (SEFIP-work) was used to measure work-related disability and pain. This instrument consists of 14 items, and each item is related to a body part. There are five possible answers for each, and the total score varies between 0 and 56 points; the higher the score, the greater the disability and pain. This instrument presents suitable measurement properties for Brazilian Portuguese [2, 16].

### Statistical analysis

Descriptive data analysis was performed to characterize the sample with the presentation of quantitative variables through mean and standard deviation and qualitative variables through absolute number and percentage.

Regarding the validity of the internal structure, the structure of the WRFQ 2.0 with five domains proposed by Abma et al. [9] was tested by means of the confirmatory factor analysis (CFA) using the R Studio software (Boston, MA, USA) and the lavaan and semPlot packages. CFA was performed with the implementation of a polychoric matrix and the robust diagonally weighted least squares (RDWLS) extraction method. Model fit was assessed by the following indices: root mean square error of approximation (RMSEA) with a 90% confidence interval (CI), comparative fit index (CFI), Tucker-Lewis index (TLI), standardized root mean square residual (SRMR), and chi-square/degrees of freedom (DF). Values > 0.90 were considered adequate for CFI and TLI, and values < 0.08 were considered adequate for RMSEA and SRMR. Values < 3.00 were considered adequate in the interpretation of the chi-square/DF [17, 18]. In the CFA, factor loadings ≥ 0.40 were considered adequate for the domain.

The internal consistency of the questionnaire was calculated using Cronbach's alpha to identify whether there were redundant or heterogeneous items in the questionnaire. Cronbach's alpha values considered in adequate range between 0.70 and 0.95 [19].

Reliability test–retest was measured using the ICC. The interpretation of the ICC value was based on the study by Fleiss [20], with values > 0.75 being accepted as adequate. In addition, standard error of measurement (SEM) and minimum detectable difference (MDD) were also calculated. Mathematical formulas for calculating SEM and MDD are described in the study by Bassi et al. [21].

To determine the construct validity after analyzing the data distribution using the Kolmogorov–Smirnov test, the Spearman correlation coefficient (rho) was used to determine the magnitude of the correlation between the WRFQ 2.0 and the WAI, NPRS and SEFIP-work. Our hypothesis was that the correlations are significant and with a magnitude < 0.30 with the instruments used in this study, given that they are an unrelated construct [11]. The significance level adopted for the hypothesis tests used in this study was 5%.

## Results

During the translation phase, the Brazilian version of the WRFQ 2.0 underwent only one cross-cultural adaptation. In Brazil, pounds are not a usual unit for mass. Item 11 referred to 10 pounds, which equated to approximately 4.53 kg. The expert committee decided to round it up to 5 kg as they thought it was more intuitive. The pre-final version of the WRFQ 2.0 was applied to 30 Brazilian workers and there was 100% comprehension of all 27 items in the questionnaire. The final version of WRFQ 2.0 is available in Additional file 1.

Regarding the sample, 220 workers were initially recruited for the study, with 26 workers evaluated in person and 194 collected through an online platform. From this sample, 23 workers who completed the online form were excluded from the study for being unemployed or on medical leave, leaving a final sample of 197 participants. The characterization of the sample is described in Tables 1 and 2. We observed that the majority of the workers were young adults, female, slightly overweight, physical activity practitioners and non-smokers.

**Table 1:** Descriptive analysis of personal and occupational characteristics of a quantitative nature

Characteristics	Mean (standard deviation)
Age (years)	37.47 (10.62)
Mass (kg)	72.37 (16.23)
Height (m)	1.66 (0.08)
Body mass index (kg/m^2^)	25.86 (4.63)
Time of work at the same job (months)	109.51 (102.11)
Working hours	33.68 (15.09)
Numerical Pain Rating Scale (score, 0–10)	3.75 (2.89)
Work Ability Index	
Domain 1 (score, 0–10)	8.07 (1.42)
Domain 2 (score, 2–10)	7.92 (1.44)
Domain 3 (score, 1–7)	3.72 (2.34)
Domain 4 (score, 1–6)	5.45 (0.73)
Domain 5 (score, 1–5)	4.50 (0.77)
Domain 6 (score, 1–7)	6.36 (1.56)
Domain 7 (score, 1–4)	3.04 (0.77)
SEFIP-work (score, 0–56)	4.19 (4.66)
Work Role Functioning Questionnaire 2.0	
Domain 1 (score, 0–100)	71.25 (24.17)
Domain 2 (score, 0–100)	73.87 (21.73)
Domain 3 (score, 0–100)	71.70 (24.37)
Domain 4 (score, 0–100)	75.45 (20.93)
Domain 5 (score, 0–100)	75.20 (22.80)

Work Ability Index, Domain 1: current work ability compared to the best in life, Domain 2: ability to work in relation to the demands of the job, Domain 3: number of current diseases diagnosed by a physician, Domain 4: estimated loss to work due to illness, Domain 5: absenteeism due to illnesses in the last year, Domain 6: proper prognosis of work ability in 2 years, Domain 7: mental resources; SEFIP-work: Self-Estimated Functional Inability because of Pain questionnaire for workers; Work Role Functioning Questionnaire 2.0, Domain 1: work scheduling demands, Domain 2: output demands, Domain 3: physical demands, Domain 4: mental and social demands, Domain 5: flexible demands

**Table 2:** Descriptive analysis of personal and occupational characteristics of a categorical nature

Characteristics	Number (%)
Gender	
Female	128 (65%)
Male	69 (35%)
Marital status	
Single	89 (45.2%)
Married	91 (46.2%)
Divorced	17 (8.6%)
Scholarity	
Complete primary level	1 (0.5%)
Incomplete secondary level	1 (0.5%)
Complete secondary level	31 (15.7%)
Incomplete higher education	25 (12.7%)
Complete higher education	44 (22.3%)
Incomplete postgraduate	10 (5.2%)
Complete postgraduate	85 (43.1%)
Physical activity	
Yes	106 (53.8%)
No	91 (46.2%)
Posture at work	
Standing	33 (16.8%)
Sitting	68 (34.5%)
Standing/sitting	93 (47.2%)
Standing/sitting/lying down	3 (1.5%)
Type of work	
Manual	54 (27.5%)
Non-manual	22 (11.2%)
Both	106 (53.8%)
Others	15 (7.5%)
Smoking	
Yes	7 (3.5%)
No	190 (96.5%)

**Fig. 1:**
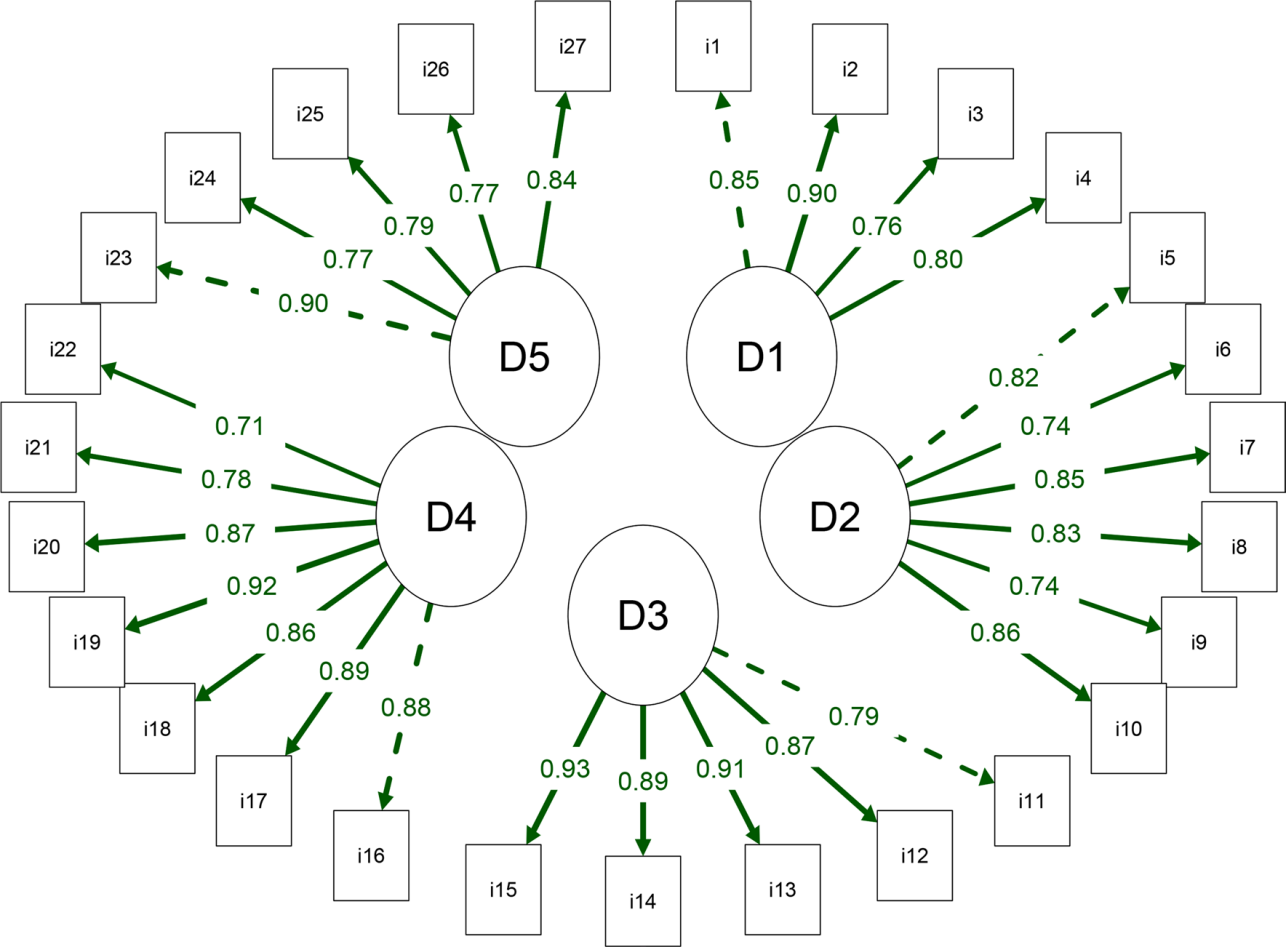
Path diagram of the internal structure of the Work Role Functioning Questionnaire 2.0 with 5 domains and 27 items. D1: work scheduling demands, D2: output demands, D3: physical demands, D4: mental and social demands, D5: flexible demands. The dotted line indicates the first item in the domain. The lines with greater thickness have greater factor loading

Based on the CFA, the structure proposed in the original WRFQ 2.0 study with five domains and 27 items presented adequate fit indices for the Brazilian version of the questionnaire: chi-square/DF = 2.17, CFI = 0.962, TLI = 0.957, RMSEA (90% CI) = 0.077 (0.070, 0.085), and SRMR = 0.065. Furthermore, as shown in Fig. 1, an adequate factor loading (> 0.40) was observed in the relationship between the domains and items. In addition, Table 3 shows the correlation between the WRFQ domains, with correlations > 0.50 observed.

**Table 3:** Correlation between Work Role Functioning Questionnaire 2.0 domains according to the confirmatory factor analysis

Domains	Domain 1	Domain 2	Domain 3	Domain 4	Domain 5
Domain 1	–	–	–	–	–
Domain 2	0.873	–	–	–	–
Domain 3	0.701	0.605	–	–	–
Domain 4	0.754	0.812	0.623	–	–
Domain 5	0.713	0.850	0.595	0.900	–

Domain 1: work scheduling demands, Domain 2: output demands, Domain 3: physical demands, Domain 4: mental and social demands, Domain 5: flexible demands

As there is no instrument in Brazilian Portuguese that assesses the same domains as the WRFQ 2.0, we used instruments with unrelated constructs. Therefore, as expected, when the correlations of the five domains of the WRFQ 2.0 with the WAI, NPRS and SEFIP-work were performed, the magnitudes of the significant correlations varied between 0.145 and 0.338, as shown in Table 4.

**Table 4:** Correlation between the domains of the Work Role Functioning Questionnaire 2.0 and the other instruments used in this study

Instruments	Work Role Functioning Questionnaire 2.0				
	Domain 1	Domain 2	Domain 3	Domain 4	Domain 5
NPRS	− 0.193*	− 0.091	− 0.119	− 0.029	− 0.031
WAI					
Domain 1	0.298*	0.329*	0.154*	0.246*	0.255*
Domain 2	0.275*	0.277*	0.189*	0.227*	0.253*
Domain 3	0.163*	0.051	0.222*	0.164*	0.102
Domain 4	0.338*	0.198*	0.279*	0.150	0.159*
Domain 5	0.218*	0.162*	0.156*	0.158*	0.153*
Domain 6	0.097	0.098	0.145*	0.064	0.106
Domain 7	0.281*	0.304*	0.082	0.249*	0.204*
SEFIP-work	− 0.272*	− 0.191*	− 0.244*	− 0.155*	− 0.150*

Work Role Functioning Questionnaire 2.0, Domain 1: work scheduling demands, Domain 2: output demands, Domain 3: physical demands, Domain 4: mental and social demands, Domain 5: flexible demands; NPRS: Numerical Pain Rating Scale; Work Ability Index, Domain 1: current work ability compared to the best in life, Domain 2: ability to work in relation to the demands of the job, Domain 3: number of current diseases diagnosed by a physician, Domain 4: estimated loss to work due to illness, Domain 5: absenteeism due to illnesses in the last year, Domain 6: proper prognosis of work ability in 2 years, Domain 7: mental resources; SEFIP-work: Self-Estimated Functional Inability because of Pain questionnaire for workers

*Significant correlation (*p* < 0.05, Spearman correlation coefficient)

The test–retest reliability of the WRFQ 2.0 domains ranged from substantial to excellent (ICC ≥ 0.785) and the internal consistency was adequate (Cronbach's alpha ≥ 0.852), as shown in Table 5.

**Table 5:** Reliability and internal consistency of the Work Role Functioning Questionnaire 2.0 (WRFQ 2.0)

WRFQ 2.0	Test	Retest	ICC (95% CI)	SEM	SEM (%)	MDD	MDD (%)	Cronbach’s alpha
Domain 1	53.57 (20.91)	54.46 (21.25)	0.930 (0.886. 0.964)	5.58	10.33	15.46	28.62	0.852
Domain 2	59.28 (22.93)	57.85 (23.03)	0.923 (0.853. 0.960)	6.38	10.89	17.68	30.18	0.886
Domain 3	50.71 (27.52)	50.14 (26.05)	0.920 (0.848. 0.959)	7.43	14.74	20.60	40.86	0.924
Domain 4	54.18 (23.28)	44.18 (19.21)	0.802 (0.642. 0.895)	9.50	19.32	26.34	53.55	0.924
Domain 5	58.14 (23.85)	52.14 (24.07)	0.785 (0.615. 0.886)	11.11	20.15	30.79	55.85	0.909

ICC, Intraclass correlation coefficient; CI, Confidence interval; SEM, Standard error of measurement; MDD, Minimum detectable difference; Domain 1: work scheduling demands, Domain 2: output demands, Domain 3: physical demands, Domain 4: mental and social demands, Domain 5: flexible demands

## Discussion

The present study observed that the cross-culturally adapted version of the WRFQ 2.0 into Brazilian Portuguese presents: (1) an internal structure with five domains and 27 items, (2) adequate reliability and internal consistency, and (3) acceptable correlations with instruments with suitable measurement properties for Brazilian Portuguese.

Regarding the internal structure of the WRFQ 2.0, our study found the same structure proposed by the authors of the original publication of this questionnaire: five domains and 27 items. In the general working population, the original version of the WRFQ 2.0 identified adequate fit indices for the five domain internal structure (CFI = 0.981, RMSEA = 0.068) [9]. Our study found a lower value for CFI (0.962) and a higher value for RMSEA (0.077); however, both were adequate. In addition, we used other fit indices (chi-square/DF = 2.17, TLI = 0.957, and SRMR = 0.065). The Persian version found the four-factor solution [8] and a study with cancer patients found both the four and five-factor solution [22].

Regarding the correlations between the instruments, the present study showed magnitudes of significant correlations ranging between 0.145 and 0.338, which are suitable for correlations between instruments with unrelated constructs. Slightly higher values were found by Abma et al. [5] when correlating the WRFQ with the WAI (correlation magnitude ranging from 0.199 to 0.536). The Norwegian, Danish [7] and Persian versions [8] did not investigate the construct validity.

The relatively low correlations found in the present study and in the study conducted by Abma et al. [5] are explained by the fact that the WRFQ 2.0 domains are not strongly related to any other instrument, since the domains measure work scheduling demands, output demands, physical demands, mental and social demands and flexible demands. Furthermore, the COSMIN establishes that for unrelated constructs (as in the case of the present study) the expected correlation magnitude is less than 0.30 [11].

Regarding reliability, we found ICC values ranging from 0.785 to 0.930. The pioneering study of the WRFQ 2.0 found lower ICC values, ranging from 0.29 to 0.82 [5]. The Persian version found slightly higher values (ICC ranging from 0.87 to 0.96) [8]. The Norwegian and Danish version did not check reliability [7].

In Brazilian Portuguese, the WRFQ has been available for use since 2007. However, WRFQ 2.0 presents five new items formulated to reflect the changes in the nature of work in recent decades: multi-tasking, development of complementary skills, and increased delegation and autonomy of workers [5]. In this way, the WRFQ 2.0 is an updated tool that is coherent with the reality of workers, in addition to not being restricted to a specific professional class, i.e., it can be used by doctors, nurses, physiotherapists, physical education professionals and others.

In complement, Abma et al. [5] highlight the relevance of the WRFQ 2.0 in the occupational context as it allows measuring the work functioning. This measure is needed that go beyond the simple dichotomy of working versus nonworking, but that assess how workers function at work. The WRFQ 2.0 can be used to evaluate interventions aimed at work rehabilitation and the management and prevention of work disability, and to monitor how health problems impact on work functioning [5].

This study has limitations. The samples were collected in different manners, i.e., face-to-face and online; even though a recent study demonstrates similarities in these forms of data collection [23], possible doubts of the participants while filling in the assessment instruments were not clarified in the online data collection. Furthermore, a large part of the sample was collected via an online platform; consequently, no face-to-face occupational assessments were carried out on the research participants. Our sample consisted of the general working population and the analysis of measurement properties of the WRFQ 2.0 for a population with specific diseases or disorders should be considered in the future by Brazilian researchers.

## Conclusion

The Brazilian Portuguese version of the WRFQ 2.0 presents valid internal structure with five domains and 27 items, adequate construct based on correlations with other instruments, and acceptable test–retest reliability and internal consistency. In this way, difficulties perceived by workers in meeting work demands, given their physical or emotional health, can be consistently evaluated in the context of occupational health by means of the WRFQ 2.0.

## Supplementary Information

**Additional file 1**. Brazilian version of the Work Role Functioning Questionnaire 2.0.
