# Supplementary material for: Translation, cross-cultural adaptation and validation of the Work Role Functioning Questionnaire 2.0 into Brazilian Portuguese in a general population
Source: Health Qual Life Outcomes. 2022 Feb 3;20:17. doi: 10.1186/s12955-022-01924-y (PMC8812155; doi:10.1186/s12955-022-01924-y)
Supplement: Supplementary file 1 — Additional file 1. Brazilian version of the Work Role Functioning Questionnaire 2.0. [file 12955_2022_1924_MOESM1_ESM.pdf]

Work Role Functioning Questionnaire 2.0

SEU TRABALHO E SAÚDE

Estas questões pedem que você avalie a **quantidade de tempo** durante as **últimas quatro semanas** em que teve dificuldade para executar certas tarefas no seu trabalho.

A quantidade de tempo diz respeito às horas em que você trabalhou nas últimas quatro semanas.

Marque a caixa "Não se aplica ao meu trabalho" apenas se a questão descrever algo que <u>não</u> faz parte do seu trabalho.

Nas últimas <u>quatro semanas</u>, por quanto tempo durante o seu trabalho a sua saúde física ou problemas emocionais dificultaram que você fizesse o seguinte:

**Eu encontrei dificuldade para…**	Sempre (100%)	Muitas vezes	Metade das vezes (50%)	Algumas vezes	Nenhuma das vezes (0%)	Não se aplica ao meu trabalho
1. começar tranquilamente o meu dia de trabalho.	☐	☐	☐	☐	☐	☐
2. começar a trabalhar assim que cheguei ao trabalho.	☐	☐	☐	☐	☐	☐
3. trabalhar sem ter que parar para intervalos ou descanso.	☐	☐	☐	☐	☐	☐
4. manter a rotina ou cronograma.	☐	☐	☐	☐	☐	☐
5. trabalhar rápido o suficiente.	☐	☐	☐	☐	☐	☐
6. finalizar o meu trabalho a tempo.	☐	☐	☐	☐	☐	☐
7. fazer meu trabalho sem cometer erros.	☐	☐	☐	☐	☐	☐
8. satisfazer as pessoas que supervisionam meu trabalho.	☐	☐	☐	☐	☐	☐
9. me sentir realizado(a) no meu trabalho.	☐	☐	☐	☐	☐	☐
10. sentir que fiz o que sou capaz de fazer.	☐	☐	☐	☐	☐	☐
11. levantar, carregar ou mover objetos que pesavam mais de 5 kg.	☐	☐	☐	☐	☐	☐
12. sentar, ficar de pé ou permanecer na mesma posição por mais de 15 minutos enquanto trabalhava.	☐	☐	☐	☐	☐	☐

	Nas últimas <u>quatro semanas</u>, por quanto tempo durante o seu trabalho a sua saúde física ou problemas emocionais dificultaram que você fizesse o seguinte:						
	**Eu encontrei dificuldade para…**	Sempre (100%)	Muitas vezes	Metade das vezes (50%)	Algumas vezes	Nenhuma das vezes (0%)	Não se aplica ao meu trabalho
13.	repetir muitas vezes os mesmos movimentos durante o trabalho.	☐	☐	☐	☐	☐	☐
14.	me curvar, girar ou me esticar enquanto trabalhava.	☐	☐	☐	☐	☐	☐
15.	usar ferramentas ou equipamentos (por exemplo, telefone, caneta, teclado, mouse, furadeira, lixadeira ou secador de cabelo).	☐	☐	☐	☐	☐	☐
16.	manter o foco no trabalho.	☐	☐	☐	☐	☐	☐
17.	trabalhar com cuidado.	☐	☐	☐	☐	☐	☐
18.	me concentrar no trabalho.	☐	☐	☐	☐	☐	☐
19.	trabalhar sem perder a minha linha de raciocínio.	☐	☐	☐	☐	☐	☐
20.	ler ou processar facilmente informações enquanto trabalhava.	☐	☐	☐	☐	☐	☐
21.	falar com alguém pessoalmente, em reuniões ou por telefone.	☐	☐	☐	☐	☐	☐
22.	controlar meu temperamento enquanto trabalhava.	☐	☐	☐	☐	☐	☐
23.	definir prioridades do meu trabalho.	☐	☐	☐	☐	☐	☐
24.	lidar com as mudanças no meu trabalho.	☐	☐	☐	☐	☐	☐
25.	processar as informações recebidas no prazo certo, por exemplo, e-mails.	☐	☐	☐	☐	☐	☐
26.	executar várias tarefas ao mesmo tempo.	☐	☐	☐	☐	☐	☐
27.	mostrar iniciativa no meu trabalho.	☐	☐	☐	☐	☐	☐
